# Surface acoustic wave humidity sensors based on uniform and thickness controllable graphene oxide thin films formed by surface tension

**DOI:** 10.1038/s41378-019-0075-0

**Published:** 2019-07-29

**Authors:** Xianhao Le, Yihan Liu, Li Peng, Jintao Pang, Zhen Xu, Chao Gao, Jin Xie

**Affiliations:** 1 State Key Laboratory of Fluid Power and Mechatronic Systems, Zhejiang University, 310027 Hangzhou, P. R. China; 2 MOE Key Laboratory of Macromolecular Synthesis and Functionalization, Department of Polymer Science and Engineering, Zhejiang University, 310027 Hangzhou, P. R. China

**Keywords:** Nanosensors, Electronic properties and materials, Structural properties

## Abstract

Graphene oxide (GO) is a promising candidate for humidity sensing, and the uniformity and thickness of GO films are important for the reproducibility and test signal strength of humidity sensors. In this paper, uniform and thickness-controllable GO films are first formed by the surface tension of different concentrations of GO solution and then transferred to surface acoustic wave (SAW) humidity sensors. This GO film formation and transfer process has very good repeatability and stability, as evidenced by the humidity response of the sensors. With the help of the uniform and highly oxidized GO film, the humidity sensors show a significantly high sensitivity (absolute sensitivity of 25.3 kHz/%RH and relative sensitivity of 111.7 p.p.m./%RH) in a wide test range from 10%RH to 90%RH with very little hysteresis (<1%RH). The sensors achieve good reversibility, excellent short-term repeatability and stability. Moreover, the humidity sensors also show a fast response and recovery time of <10 s.

## Carbon nanomaterials: Powering high-accuracy humidity sensors

A technique that creates uniform films of graphene oxide enables the production of humidity sensors with high accuracy and stability. Technological advancement is fueling a demand for humidity sensors, but reliably producing uniform, high accuracy sensors at a low cost is still a challenge. Jin Xie and his team from China’s Zhejiang University have now developed a surface tension method to create uniform-thickness films of graphene oxide (GO), a humidity sensing material, which can then be transferred to sensor platforms. In tests, the resulting sensors showed high sensitivity from 10% to 90% relative humidity and quick response times to changing conditions. Altering the concentration of GO solutions produces films of varying thickness. This technique may be further developed for use in applications ranging from medical, to agriculture, to consumer electronics.

## Introduction

In recent years, applications of humidity sensors have expanded from traditional industry and agriculture to medical, consumer electronics, and smart homes^1,2^. With the rapid development of the internet of things, the demand for humidity sensors is also growing. Humidity sensors not only are required to have high sensitivity, a wide test range, small hysteresis, and fast response and recovery but also need to have low cost, low energy consumption, and easy integrability^3^. Currently, research on humidity sensors is mainly based on optical^4^, resistive^5,6^, capacitive^7,8^, and acoustic resonant devices^9,10^. The capacitive and resistive devices are small, simple, and low cost, but they have the disadvantage of poor test accuracy. Although the optical devices have high test accuracy, they consume too much energy and are difficult to integrate.

As one of the acoustic resonators, the surface acoustic wave sensor (SAW) has advantages of small size, high sensitivity^11,12^, simple processing, and high test accuracy due to its quasi-digital output signal^13^. Combined with aluminum nitride (AlN) as the piezoelectric material, the fabrication of SAW devices is compatible with the Complementary Metal Oxide Semiconductor (CMOS) process^14^. Moreover, the chemical stability^15^ and hydrophobicity^16^ of AlN are beneficial to the stability of the SAW device during humidity sensing.

In addition to the sensing platform, it is also important to choose appropriate humidity-sensing materials to meet the requirements of humidity sensors with high performance. Commonly used humidity-sensing materials can be divided into ceramics^17^, semiconductors^18^, polymers^19^, and nanomaterials^20^. Recently, graphene oxide (GO) has been widely used by researchers on different humidity-sensing platforms^21–23^. In addition to their large surface-to-volume ratio as a carbon nanomaterial, GO films also have high hydrophilicity^24^ and electrical insulation^25^ due to its oxygen-containing functional groups on the surface. These features make GO films very suitable for covering the surface of SAW devices as a humidity-sensing material. Our previous work has demonstrated that an AlN SAW resonator combined with a GO film has good performance in humidity sensing^26^. For practical applications, the thickness and uniformity of the GO film should be controlled, because they will greatly affect the test signal strength^27^ of the SAW humidity sensors as well as sensor-to-sensor reproducibility^28^. Nevertheless, how to deposit a uniform and thickness-controllable GO film on the surface of a device is still a problem. Although the existing methods of drop casting^29,30^, spin coating^22^, and dip coating^2^ are simple, the uniformity and thickness of the GO films formed on devices by these methods are difficult to accurately control, especially when the piezoelectric material is hydrophobic and the surface of the piezoelectric layer is covered with electrodes. Using ink-jet printing^23^, spray coating^31^, and ultrasonic atomization^32^, the thickness of GO films can be controlled, but the obtained GO films still have poor uniformity. In summary, because of the limitation of the surface structure of the SAW device and the hydrophobicity of the piezoelectric material, it is difficult to directly deposit a uniform and thickness-controllable GO film on the surface of the AlN SAW device.

Inspired by a fabrication process of free-standing inorganic sheets^33^, we propose a simple and convenient GO film-forming method based on the surface tension of GO solution by using copper rings in this paper. When the water in the GO solution evaporates, uniform GO films are gradually formed and attached to the copper rings. The thickness of the GO films can be controlled by the concentration of the GO solution. Good uniformity and thickness controllability of the GO films can be achieved by this method. After the GO films are transferred to the surface of the SAW devices, the performances of the SAW humidity sensors are investigated systematically, including their sensitivity, sensor-to-sensor reproducibility, sensing mechanism, hysteresis characteristic, stability, reversibility, repeatability, response, and recovery time.

## Materials and methods

### SAW resonators design and fabrication

**Fig. 1:**
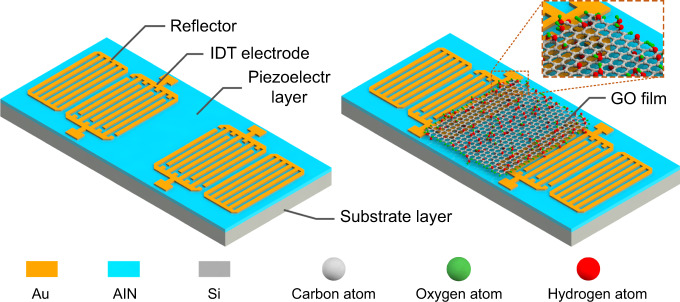
Schematic diagram of GO film based AlN SAW humidity sensor. Schematic views of the AlN surface acoustic wave sensor with a clean surface covered with a GO film

Schematics of the SAW humidity sensor before and after being covered with a GO film are illustrated in Fig. 1. The two-port SAW resonator consists of two interdigitated transducers (IDTs) and two reflectors. Each IDT has 60 pairs of interdigital electrodes, while each reflector has 120 shorted grating electrodes. The wavelength, distance between the two IDTs, and distance between one of the IDTs and one of the reflectors of the device are 20, 605 and 7.5 µm, respectively. To eliminate unwanted spurious responses^34^, the *W*/*W*_0_ electrode structure was used. *W* and *W*_0_ are acoustic apertures and total IDT apertures and the lengths of *W* and *W*_0_ are 62*λ* and 65*λ*, respectively. An AlN/Si layered structure was adopted to fabricate the SAW resonator to achieve good thermal stability^15^. After 1 µm AlN was deposited over the Si wafer by reactive sputtering, a 200 nm gold layer was patterned on the surface of the AlN layer to form the IDTs and reflectors through a lift-off process.

### GO film formation and transfer

**Fig. 2:**
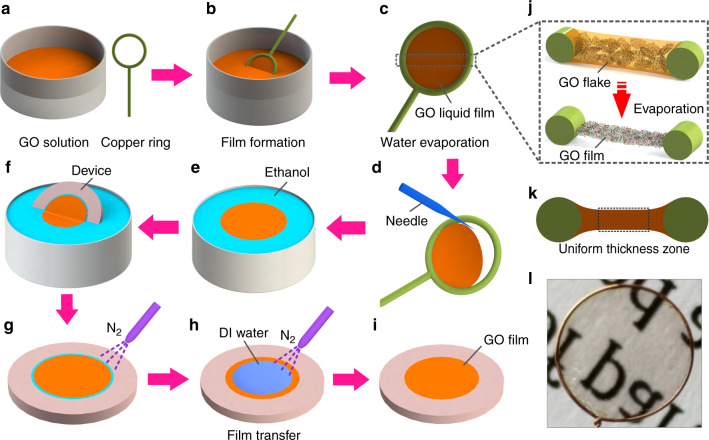
Schematic drawing of the GO film formation and transfer process. **a** preparing a copper ring and GO solution; **b** forming a GO solution liquid film; **c** drying the GO film; **d** releasing the GO film from the copper ring; **e** transferring GO film to ethanol; **f** transferring the GO film to the device; **g** drying the GO film with nitrogen; **h** flattening and drying the GO film; **i** device with transferred GO film. **j** Detailed process of changing a GO solution liquid film to a dried GO film. **k** Sketch of the thickness distribution of the GO solution liquid film on the copper ring. **l** Optical image of a formed GO film

The processes of GO film formation and transfer started with GO aqueous dispersions and copper rings, as shown in Fig. 2a. The GO solution with an original concentration of 2 mg/ml was prepared by the modified Hummers method^35,36^ and the copper rings with a 4.5 mm diameter were made of 0.1 mm diameter copper wire. To achieve the formation of different thicknesses of GO films, some of the original solution was further diluted to 1.5 and 1 mg/ml with deionized water. When the copper rings in the solutions were slowly removed, the GO solutions became liquid films adhered to the copper rings due to the surface tension of the solutions (Fig. 2b, c). GO films with different thicknesses were gradually formed after the water in the GO solutions with different concentrations evaporated. The detailed formation process of dried GO films from the GO solution liquid film is illustrated in Fig. 2j. GO is rich in carboxyl functional groups, which give it excellent dispersibility in water^37^. Therefore, in the liquid films, GO nanosheets were also uniformly distributed. Furthermore, GO can reduce the surface tension of the liquid films such as a surfactant^38^, so the GO solution liquid films can expand to a large size in the copper rings. During the gradual evaporation of the water in the liquid films, the GO nanosheets were self-assembled into GO films by forming *π*–*π* stacking interactions and hydrogen bonds^39^. After drying for ~ 2 h at room temperature, the formed GO films were ready to be transferred to devices. First, a needle was used to pierce the GO films and release them from the copper rings (Fig. 2d), and then the GO films were transferred to an alcohol solution (alcohol has a small surface tension relative to water, making it easier to transfer small-sized GO films to the devices) with tweezers and allowed to float on the surface (Fig. 2e). Afterwards, the devices were immersed in alcohol and the films were slowly picked up from the bottom (Fig. 2f). To obtain flat GO films on the surface of the devices, the films were blown dry with N_2_ (Fig. 2g). After that, drops of deionized water were dropped on the films and then blown dry to increase the flatness and adhesion of the films (Fig. 2h). Finally, devices with uniform GO films were obtained (Fig. 2i).

### Characteristics of GO films and SAW humidity sensors

**Fig. 3:**
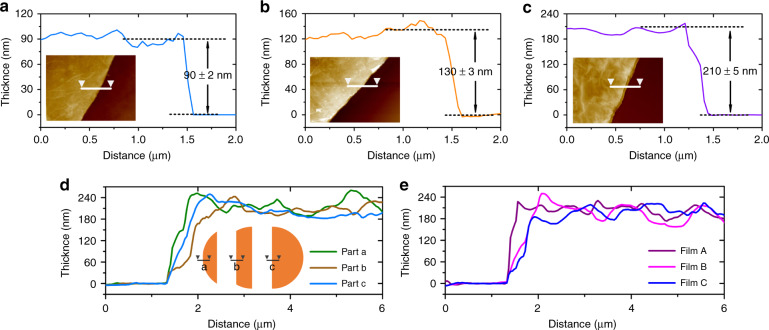
Thickness calibration of GO films. **a**–**c** AFM images and profile plots of the GO films formed by GO solutions with different concentrations. **d** Thickness profiles of the same GO film at different parts. **e** Thickness repeatability of the GO films formed by GO solutions with the same concentrations

As the diameter of the copper ring is much larger than the diameter of the copper wire, the thickness of the GO solution liquid film attached to the copper ring is thinned by the surface tension. The thickness distribution of the liquid film is depicted in Fig. 2k, except for the area close to the copper ring; the figure shows that the liquid film has a relatively uniform thickness in most areas. GO nanosheets can be uniformly distributed in a liquid film so that a GO solution liquid film in a uniform thickness zone can be dried to obtain a GO film with uniform thickness, as shown in Fig. 2l. The homogeneous color in most areas of the GO film indicates the uniform thickness of the GO film and the uniform thickness area is large enough to cover the surface of the tested device. Different thickness GO films can be obtained by changing the concentration of the GO solution. Figure 3a–c show AFM images and height profiles of the partial GO films formed by different concentrations (1, 1.5, and 2 mg/ml) of GO solution. The thicknesses of the obtained GO films are 90, 130, and 210 nm, respectively. As the Atomic Force Microscope (AFM) can only be used to measure the edge portion thickness of the GO film, we further divided the film into three parts to test the inner portion thickness of the film. As shown in Fig. 3d, the average thickness of the entire GO film is kept at 210 ± 10 nm. Figure 3e compares the thickness of the GO films formed by GO solutions with the same concentration; the average thickness variance of the GO films is approximately ± 15 nm. The repeatability of the film thickness is relatively good, excluding the influence of test error.

**Fig. 4:**
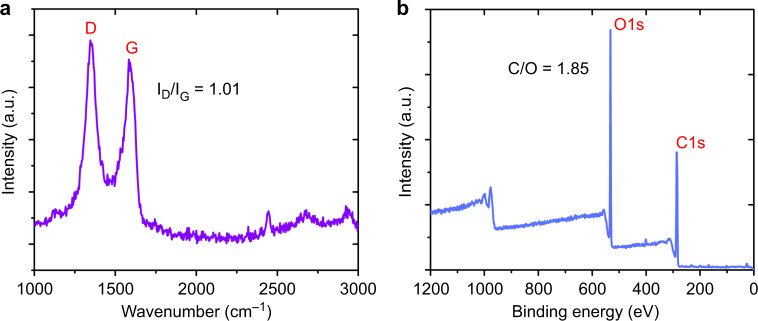
GO characteristics. **a** Raman spectrum of GO. **b** XPS survey scan of GO

Increasing the oxidation degree of GO can improve its water absorption performance^40^, thereby improving the sensitivity of a GO-based humidity sensor. For this reason, the GO selected in this work was highly oxidized, as evidenced by the Raman spectrum and X-ray Photoelectron Spectroscopy (XPS) survey scan of GO in Fig. 4a, b. The two distinct peaks at 1349.88 and 1583.26 cm^−1^ in the Raman spectrum correspond to the well-known D and G bands, respectively, and the band intensity ratio, *I*_D_/*I*_G_, can be used as an indicator for evaluating the oxidation degree of GO^41^. The higher the *I*_D_/*I*_G_ ratio is, the higher the degree of oxidation (the *I*_D_/*I*_G_ ratio is 1.01 for the selected GO). In addition, the C/O ratio in GO can also be used to measure the degree of oxidation of GO^42^. Highly oxidized GO typically has a lower C/O ratio and the calculated C/O ratio of selected GO is 1.85.

**Fig. 5:**
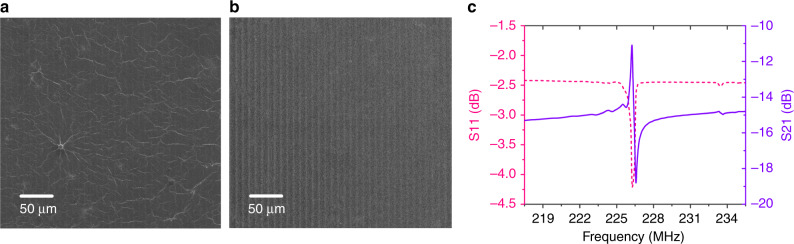
Comparison of the uniformity of GO films prepared by different methods. SEM images of SAW resonators with GO films formed by drop casting (**a**) and surface tension (**b**). **c** Transmission spectrum (S_11_ and S_21_) of the SAW device with a GO film

**Fig. 6:**
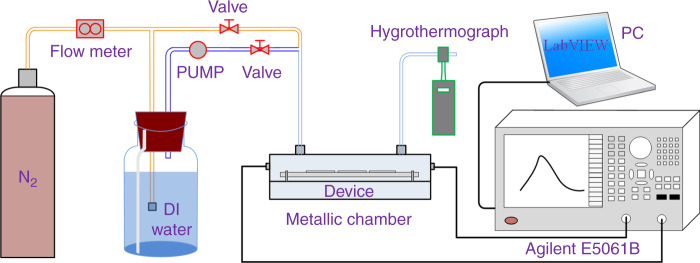
Experimental schematic diagram of the humidity measurement system

Compared with the GO film deposited directly on the surface of the SAW device by the drop-casting method, the film obtained by the proposed surface-tension method has obvious advantages in uniformity and smoothness, as displayed in Fig. 5a, b. Figure 5c plots the typical transmission spectra (S_11_ and S_21_) of the SAW resonator with a GO film. The resonant frequency of the sensor is ~226.3 MHz according to the peak position on the spectrum curve. During the test, we can track the peak position to reflect the resonant frequency change of the sensor. To study the stability of the GO film prepared by the surface-tension method and sensing mechanism of the humidity sensor, we prepared six sensor samples and the sensor characteristics are summarized in Table 1. Sensors D1, D2, and D3 consisted of the same SAW resonators and GO films formed by GO solutions with the same concentration, whereas GO films prepared by GO solutions with different concentrations were adopted in sensors D1, D4, and D5. For sensor D6, the selected SAW resonator had a metallized path, which was used to eliminate the influence of GO film conductivity changes on the sensor response. The sensors with GO films were kept in an electronic moisture-proof box for a few weeks before humidity testing, as the structure and chemical properties of the GO film need a period of time (~1 month) to become stable^43^. During the humidity test, the sensors were fixed successively in a relatively sealed metallic chamber, as shown in Fig. 6. By changing the flow rate of the dry and wet N_2_ into the chamber, the humidity inside the chamber can be adjusted (5% RH to 95% RH), whereas the humidity and temperature inside the box were calibrated in real time through a commercial hygrothermograph (TASI-621) connected to the outlet of the chamber. A network analyzer (E5061B) together with a PC with the LabVIEW program was used to detect and record changes in the resonant frequencies of the sensors.

**Table 1:** Summary of the characteristics of the SAW humidity sensors

Sample	Surface configurations	Resonant frequency (MHz)	GO thickness (nm)
D1	Bare path	226.3	210
D2	Bare path	226.3	210
D3	Bare path	226.3	210
D4	Bare path	226.3	130
D5	Bare path	226.3	90
D6	Metallized path	221.2	210

## Results and discussion

### Humidity-sensing performance and sensing mechanism

**Fig. 7:**
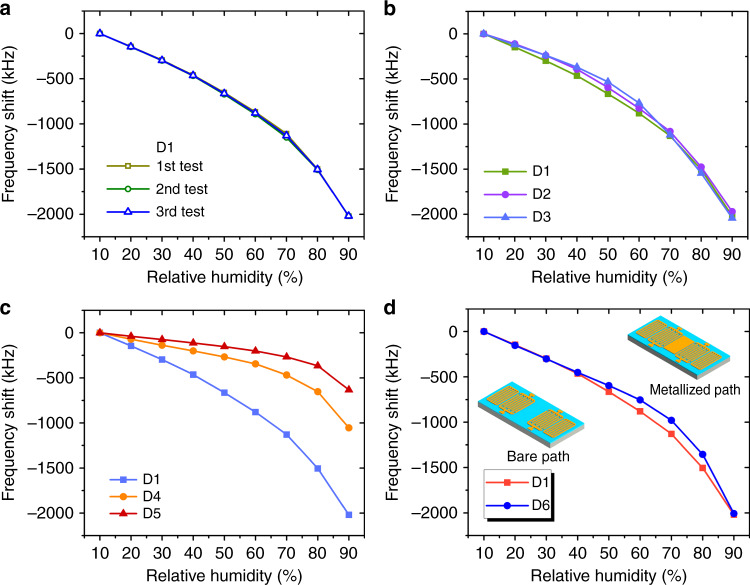
Static humidity response test of GO film based AlN SAW humidity sensors. **a** Frequency shifts as a function of relative humidity for the same sensor under repeated testing. **b** Sensing performance of the sensors with GO films formed by the same copper ring and same concentration of GO dispersion. **c** Sensing performance comparison of the sensors with different thickness GO films (**d**) and different surface configurations of the SAW resonators

The humidity-sensing performance of sensor D1 was first tested repeatedly from 10%RH to 90%RH with an interval of 10%RH. Figure 7a shows the relationship between the resonant frequency shifts of sensor D1 and the relative humidity. The frequency shifts of the sensor obtained from the three repeated tests (every 3 days) are almost the same at each humidity stage, which indicates that the performance of the GO film covering the sensor surface is stable. Subsequently, the stability of the GO film formation and transfer method was investigated by comparing the sensing performance of sensors D1, D2, and D3. Very small differences between the responses of the sensors to humidity changes were observed, as evidenced by Fig. 7b. These test results indicate that the GO film formation using surface tension and transfer processes have good stability and repeatability, demonstrating the great potential of this approach for commercial humidity sensor processing.

During the humidity test, AlN and Si do not absorb water, and only the GO film absorbs water. The changes in the resonant frequency of the sensors are mainly caused by changes in the mass, viscoelasticity, and conductivity of the GO films after water moisture absorption^44^. GO films with different thicknesses have different adsorption capacities for water moisture, so the thickness of the GO films is one of the factors affecting the sensing performance of the sensors. Figure 7c compares the sensing performance of sensors D1, D4, and D5 with different thicknesses of GO films. The sensor with a thicker GO film has better sensing performance, because the thicker GO film absorbs more water moisture during the test, resulting in a larger frequency shift change of the sensor. Although the thickness of the GO film can be several hundred nanometers, it is negligible compared with the thickness of the SAW (1 to 2 wavelengths) propagating on the sensor substrate. Moreover, the shear modulus of the GO film is large^45^. For this reason, the GO film can be regarded as an acoustic thin film^46^ and the viscoelastic change in the GO film has little effect on sensor response. In addition, the conductivity of the GO film increases with increasing humidity, but as the frequency of the test signal increases, the magnitude of the change in the conductivity of the GO film gradually decreases^47^. SAW devices usually operate at frequencies of a few hundred megahertz and the conductivity of the GO film should be insensitive to humidity changes at such high frequencies. To further verify the effect of GO film conductivity changes on the sensor response, the sensing performance of sensor D6 with a metallized path was tested and compared with that of sensor D1. As shown in Fig. 7d, the change in GO film conductivity does not contribute much to the response of the sensor. Based on the above analysis, the mass change in the GO film is the main factor causing the resonant frequency change of the sensor. According to Sauerbrey’s equation^48^, the relation between the mass change of the GO film (Δ*m*) and the resonant frequency shift of the sensor (Δ*f*) can be described as:

1 $$\Delta f = - Cf_0^2\Delta m/A$$

where *f*_0_ is the resonant frequency, *C* is a constant, and *A* is the sensing area. There is a linear relationship between the mass change and the resonant frequency shift according to the above equation. However, as shown by the humidity response curves of all the sensors, the resonant frequency shifts increase nonlinearly with increasing relative humidity, which means that the mass increases of GO films are nonlinear during this process. This phenomenon is because at high humidity levels, the water molecules gradually penetrate into the interlayer of the GO film and enlarge the layer spacing of the film^39^, thereby increasing the water absorption capacity of the film, resulting in a greater increase in the mass change. The sensing performance of the sensors is quantified as absolute sensitivity (*S*_a_) and relative sensitivity (*S*_r_) for easy comparison (*S*_a_ = Δ*f/*Δ*RH*,*S*_r_ = *S*_a_/*f*_0_, Δ*RH* is change in relative humidity), and the absolute sensitivity and relative sensitivity of our sensors reach up to 25.3 kHz/%RH and 111.7 p.p.m./%RH. Compared with other SAW and QCM humidity sensors based on different sensing materials summarized in Table 2, the humidity sensors in this work have obvious advantages in relative sensitivity.

**Table 2:** Summary of the characteristics of different humidity sensors

Reference	Sensor type	Sensing material	Fabrication method	*S*_*r*_ (p.p.m./%RH)	Response and recovery time (s)
This work	SAW	GO (210 nm)	Surface tension	~ 111.7	10, 9
30	SAW (ZnO/polyimide)	GO (400 nm)	Drop casting	~ 88.9	22, 5
32	SAW (Quartz)	GO (~70 nm)	Atomization	~ 2.5	NA
12	SAW (AlN/Si)	Ga-doped ZnO (300 nm)	Spin coating	~ 37.5	NA
11	SAW (LiNbO_3_)	Polyaniline nanofibres	Drop casting	~ 24.3	NA
10	Quartz Crystal Microbalance (Quartz)	GO (126 nm)	Spin coating	~ 3.0	18, 12
6	Resistance	PDDA/RGO	LbL self-assembly	~ 3857	108, 94
8	Capacitance	GO	Drop casting	~ 4725000	10.5, 41
TASI-621	Capacitance (commercial)	NA	NA	NA	60

### Hysteresis, stability, and repeatability

**Fig. 8:**
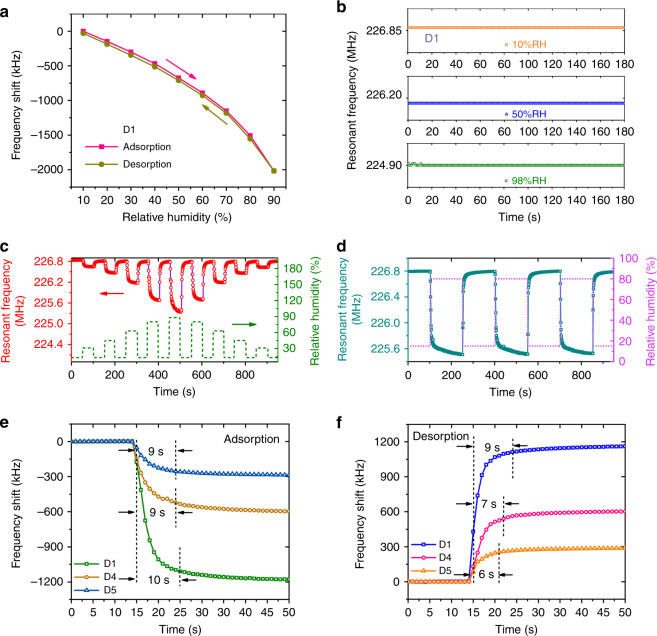
Dynamic humidity response test of GO film based AlN SAW humidity sensors. **a** Humidity hysteresis characteristic of sensor D1. **b** Fluctuation in the resonant frequency of sensor D1 at fixed humidity levels. **c** Continuous response of the resonant frequency of sensor D1 to different humidity range changes. **d** Short-term repeatability of sensor D1 when the relative humidity is repeatedly changed between 15%RH and 80%RH. Detailed response (**e**) and recovery (**f**) processes of the sensors

As a humidity-sensing layer, the GO film can quickly absorb and desorb water molecules, thus allowing the humidity sensors to have low hysteresis. Among all the sensors, sensor D1 was used to study hysteresis, because it has the thickest GO film. The humidity hysteresis characteristic of sensor D1 was tested by changing the relative humidity from 10%RH to 90%RH and then quickly back to 10%RH. In Fig. 8a, very little hysteresis (<1%RH) is observed within the entire humidity test range for the sensor with the thickest GO film, which indicates the potential applications in high humidity levels. Test stability is also very important for humidity sensors, especially for sensors that require high accuracy. Figure 8b records the resonant frequency of sensor D1 when the relative humidity is fixed at different levels (10%RH, 50%RH, and 90%RH). The sensor exhibits excellent stability at low humidity levels and only a very small variation in the resonant frequency of the sensor appears at very high humidity levels.

The continuous response of sensor D1 to humidity changes was investigated by continuously adjusting the humidity from 15%RH to different humidity levels and then back to 15%RH. The resonant frequency changes of the sensor were measured and recorded with a time interval of 1 s and the sensor shows very good tracking performance as the humidity is continuously changed between different relative humidities. When the humidity is set back to 15%RH, the sensor can quickly recover to its initial state, as indicated in Fig. 8c. The short-term repeatability of sensor D1 was further studied by repeatedly changing the humidity between 15%RH and 80%RH, as shown in Fig. 8d. Excellent sensor repeatability is obtained over three cyclic tests. At a high humidity level, the resonant frequency of the sensor continuously decreases slowly, which is mainly caused by the slow increase in the humidity in the chamber to the set value.

### Response and recovery

The response and recovery speed of sensors D1, D4, and D5 were investigated by rapidly changing the relative humidity between 15%RH and 80%RH, and detailed response and recovery processes of the sensors are shown in Fig. 8e, f. With reference to commercial humidity sensors, response and recovery time can be defined as the time from when the resonant frequency starts to change until the resonant frequency reaches 95% of its final value. According to the above definition, the response and recovery time of all sensors is < 10 s, which is much faster than that of capacitive and resistive humidity sensors, as listed in Table 2.

## Conclusion

In summary, we proposed a surface-tension method to obtain uniform and thickness-controllable GO films for use as humidity-sensing layers to improve the performance of SAW humidity sensors. The thickness of the GO films can be controlled by adjusting the concentration of GO solution. This GO film formation and transfer process has good repeatability and stability. Compared with other GO-based SAW humidity sensors, our sensors have significantly high sensitivity because of the use of highly oxidized GO. Increasing the thickness of the GO film can further improve the sensing performance of the humidity sensor, as the water absorption of the GO film is the main cause of the resonant frequency change of the sensor. Furthermore, theoretical analysis and experimental tests were carried out to investigate the effects of GO film viscoelasticity and conductivity changes on sensor response during humidity sensing, illustrating that the mass loading effect is the main contributor to the sensing mechanism of the sensor. Furthermore, very little humidity hysteresis and good stability of the sensors are obtained. The humidity sensors also show excellent reversibility and short-term repeatability, a fast response and a short recovery time.
